# Prediction of putative potential siRNAs for inhibiting SARS-CoV-2 strains, including variants of concern and interest

**DOI:** 10.2217/fmb-2021-0130

**Published:** 2022-03-14

**Authors:** Abdullah Al Saba, Maisha Adiba, Sajib Chakraborty, AHM Nurun Nabi

**Affiliations:** ^1^Department of Biochemistry and Molecular Biology, Laboratory of Population Genetics, University of Dhaka, Dhaka, 1000, Bangladesh; ^2^Department of Biochemistry and Molecular Biology, Systems Cell-Signalling Laboratory, University of Dhaka, Dhaka, 1000, Bangladesh

**Keywords:** COVID-19, novel coronavirus, SARS, SARS-CoV-2, siRNA

## Abstract

**Aim:** To predict siRNAs as a therapeutic intervention for highly infectious new variants of SARS-CoV-2. **Methods:** Conserved coding sequence regions of 11 SARS-CoV-2 proteins were used to construct siRNAs through sampling of metadata comprising 214,256 sequences. **Results:** Predicted siRNAs S1: 5′-UCAUUGAGAAAUGUUUACGCA-3′ and S2: 5′-AAAGACAUCAGCAUACUCCUG-3′ against RdRp of SARS-CoV-2 satisfied all the stringent filtering processes and showed good binding characteristics. The designed siRNAs are expected to inhibit viral replication and transcription of various coronavirus strains encompassing variants of concern and interest. **Conclusion:** The predicted siRNAs are expected to be potent against SARS-CoV-2, and following *in vitro* and *in vivo* validations may be considered as potential therapeutic measures.

Of the several bacterial and viral pathogens that humankind has encountered to date, SARS-CoV-2, caused by novel coronavirus, is the latest, triggering COVID-19 and leading to a significant number of deaths and infected people. The virus is genetically similar to SARS-CoV-1 and MERS-CoV, which are phylogenetically classified in the *Betacoronavirus* genus, but neither has caused such a vast irreparable loss of humankind and pandemic as SARS-CoV-2 [1]. This deadly virus contains an unsegmented, 3′ polyadenylated and 5′ capped positive-sense, single-stranded, ∼30-kb-long RNA genome [2]. This genome codes for 9860 amino acids and results in nonstructural and structural proteins, including ORF1ab, surface glycoprotein (S), ORF3a, envelope protein (E), membrane glycoprotein (M), ORF6, ORF7a, ORF7b, ORF8, nucleocapsid phosphoprotein (N) and ORF10 protein [3].

According to WHO, there were a total of 245,373,039 confirmed cases of COVID-19, including 4,979,421 deaths, around the world by 30 October 2021. The high mutational rate of SARS-CoV-2 has led to the generation of many variants. Certain variants, possessing specific mutations, particularly in the receptor binding domain of surface glycoprotein, are increasing the transmissibility of the virus, reducing its neutralization by antibodies generated from prior infection or vaccination, making the virus difficult to detect by diagnostics [4]. The CDC has classified B.1.1.7, B.1.351, P.1, B.1.427 and B.1.429 as ‘variants of concern’ and B.1.526, B.1.526.1, B.1.525, B.1.617, B.1.617.1, B.1.617.2, B.1.617.3 and P.2 as ‘variants of interest’ in the USA [5]. These variants harbor the mutation D614G, which has been reported to increase the transmissibility of the virus [6,7]. Variant B.1.617 (also called ‘double mutant’) and its sublineages, B.1.617.1, B.1.617.2 and B.1.617.3, have recently been recognized as ‘variants of interest’ in the USA. Although the mutations E484Q and L452R appear separately in many SARS-CoV-2 variants, both of these mutations have been found together in the variant B.1.617 [8,9]. However, the sublineage B.1.617.2 does not contain the E484Q mutation. In BNT162b2-elicited serum, neutralization titers are lower compared with B.1.1.7 and P.1 [10]. B.1.351 has more resistance to neutralization by the neutralizing antibodies elicited by the Moderna and Novavax vaccines compared with variant B.1.429 [11]. SARS-CoV-2 variants B.1.526.1, B.1.427 and B.1.429 harbor the L452R mutation, whereas B.1.525, P.2, P.1 and B.1.351 contain the E484K mutation (some B.1.526 and B.1.1.7 variants also contain the E484K mutation) [5]. K417N/T, E484K and N501Y mutations in the receptor binding domain of surface glycoprotein confer higher resistance to neutralization [12].

Apparently, vaccination is the best way to control the COVID-19 pandemic. The vaccine development process is time consuming and it can protect humans for 6–8 months after a booster vaccination by augmenting cellular immune response [13]. To date, the inactivated virus vaccine Covaxin from Bharat Biotech, India [14], viral vector vaccine Gam-COVID-Vac or Sputnik V from Russia [15] and AstraZeneca vaccine-AZD1222 (ChAdOx1) from the University of Oxford, UK [16] and RNA vaccines from Moderna (mRNA-1273) [17] and Pfizer (BNT162b2), both from USA [18] are some of the vaccines that are either approved for human use or in trial stages as preventive measures against SARS-CoV-2. However, the complex genetic makeup of novel SARS-CoV-2 and its high mutation rate make the vaccine response uncertain [19]. In addition, there are many drugs currently being tested based on previous experience preventing SARS-CoV and MERS-CoV. These drugs include protease inhibitors (lopinavir/ritonavir), nucleoside analog (ribavirin), antiparasitic drug (chloroquine), RNA polymerase inhibitor (favipiravir), adenosine analog which inhibit RdRP (remdesivir) and several natural products [20]. However, none of these are proven to be a definitive cure, and because of the rapid mutation rate of the virus along with its dynamic nature, any targeted drug is bound to take longer to develop [21].

Thus, in addition to all of these therapeutic approaches, a promising option of antiviral therapy could be based on an endogenous RNAi mechanism. RNAi refers to gene silencing at the mRNA level guided by small complementary ncRNA species [22,23]. siRNAs are double-stranded ncRNA molecules comprising 20–25 bp that can regulate the expression of genes; thus, siRNA-based therapeutics have been developed and implemented for anticancer, antiviral and genetic disease purposes [24,25]. siRNAs have the potential to reduce replication and viral infection by inserting into the cell as long dsRNAs, which are then turned into ∼21-bp small dsRNAs upon cleavage by RNase III (Dicer). This dsRNA further enters the RNA-induced silencing complex (RISC), turns into ssRNA and forms a complex that recognizes specific target mRNA sites and cleaves mRNA [20]. Therefore, in this study, the authors attempted to design siRNAs specific to various conserved regions of 11 SARS-CoV-2 proteins corresponding to 992 SARS-CoV-2 sequences so that they can inhibit translation and thus be used for therapeutic purposes against this virus.

## Methods

### Retrieval of coding sequences of SARS-CoV-2 proteins

Coding sequences (CDSs) of SARS-CoV-2 proteins were retrieved from the National Center for Biotechnology Information (NCBI) virus portal (www.ncbi.nlm.nih.gov/labs/virus/vssi/#/). The portal virus, nucleotide completeness and host fields were set to ‘severe acute respiratory syndrome coronavirus 2 (SARS-CoV-2), taxid: 2697049’, ‘complete’ and ‘*Homo sapiens* (human), taxid: 9606’, respectively, for retrieving the sequences analyzed in the present study. The collection date field was set from 1 September 2020 to 6 May 2021. The metadata (containing accession number, Pango lineage, release date, geographical location, collection date) of all the nucleotide sequences of SARS-CoV-2 fulfilling the aforementioned criteria were obtained from the same database. Sequences that did not mention the month of specimen collection were not included. Sequences with accession numbers starting with ‘FR’, ‘HG’, ‘LC’, ‘LR’, ‘OA’, ‘OB’, ‘OC’ and ‘OD’ were not included, as no annotations were provided for these sequences. Accession numbers of SARS-CoV-2 nucleotide sequences from each were selected randomly as previously mentioned [5]. Sequences were collected based on the sample collection date so that the viral strains prevalent in that month would be closely reflected in the sequences collected. The randomly selected sequences were finally retrieved from the NCBI virus portal. The sequences for which Pango lineages were not mentioned were assigned the Pango lineage using the Pangolin web server (https://pangolin.cog-uk.io/). CDSs of the 11 proteins (S, N, E, M, ORF1ab polyprotein, ORF3a, ORF6, ORF7a, ORF7b, ORF8 and ORF10) of SARS-CoV-2 corresponding to a particular nucleotide sequence were obtained from the virus portal.

### Multiple sequence alignment of the coding regions

CDSs corresponding to the selected nucleotide sequences of SARS-CoV-2 for a particular protein were aligned using the online MAFFT platform (https://mafft.cbrc.jp/alignment/server/). Default parameters were used. CDSs for all proteins were aligned similarly. In the case of ORF1ab, the CDSs for leader protein (266–805), 3C-like proteinase (10055–10972) and RdRp (13442–13468, 13468–16236) were manually extracted from the multiple sequence alignment (MSA) file of ORF1ab using the reference SARS-CoV-2 nucleotide sequence annotation, NC_045512.2. Hence, the CDSs of 13 proteins of SARS-CoV-2 were further analyzed in this study.

### Extraction of conserved regions from MSA files of CDSs

The MSA files were visualized using BioEdit 7.2.5, and 100% conserved regions for the coding regions of each of the 13 proteins (S, N, E, M, ORF3a, ORF6, ORF7a, ORF7b, ORF8, ORF10, leader protein, 3C-like proteinase and RdRp) were manually extracted. Conserved regions shorter than 21 residues were not included.

### Target-specific siRNA prediction

Conserved regions of the CDSs of the 13 SARS-CoV-2 proteins were used for designing target-specific siRNAs using the siDirect 2.0 web server (http://sidirect2.rnai.jp/). Functional siRNA selection algorithm was set to combine rule Ui-Tei + Reynolds + Amarzguioui. siRNAs that fulfilled all the three rules (Ui-Tei [26], Reynolds [27] and Amarzguioui [28]) were selected (Ui-Tei, Reynolds and Amarzguioui in functional siRNA selection column in server) for further analysis (Supplementary File 1). Maximum temperature for seed duplex target was kept at default (<21.5°C). Guanine–cytosine (GC) content was set from 30 to 64% in the siDirect 2.0 server [29].

The siRNAs predicted from siDirect 2.0 were then cross-checked with the result from i-Score Designer, which calculates scores for nine different siRNA designing algorithms: Ui-Tei [26], Amarzguioui [28], Hsieh [30], Takasaki [26], s-Biopredsi [31], i-Score and Reynolds [32], Katoh [32] and Designer of siRNA (DSIR) [33]. The target sequences of the siRNAs predicted from siDirect 2.0 were used as input in i-Score Designer for analysis. Finally, the siRNAs predicted by siDirect 2.0 that overlapped with siRNAs predicted by i-Score Designer to have a rank of 1 in all three algorithms (s-Biopredsi, i-Score and DSIR) were selected for further analysis.

### Filtering siRNAs with palindromic sequences & off-target assessment of siRNAs

Palindromic sequences were inspected in both guide and passenger strands using the EMBOSS palindrome online tool (www.bioinformatics.nl/cgi-bin/emboss/palindrome). The minimum length of the palindrome was set at four.

The off-target assessment of siRNAs was performed in three steps. First, for assessment of perfect or near-perfect homology with the unwanted transcripts, both guide and passenger strands of siRNAs were blasted against the NCBI transcript reference sequences database in NCBI BLAST (https://blast.ncbi.nlm.nih.gov/blast.cgi). BLAST parameters were changed accordingly for precise alignment with the short query sequences [29]. The BLASTN program was selected. Word size was selected at seven. Expect threshold was set at 1000 for stringent specificity check. siRNAs with query coverage ≥78% were discarded. Gap opening, gap extension and mismatch penalty were set at 2, 2 and -1, respectively. BLAST parameters were changed for precise alignment with the short query sequences. In the second step, assessment was done for partial homology (seed-dependent off-target or miRNA-like off-target). Although seed-dependent off-target had already been minimized by selecting siRNAs with melting temperature (Tm) <21.5°C, the Genome-wide Enrichment of Seed Sequence matches (GESS) online tool (www.flyrnai.org/gess/) was used for a more stringent check. Candidate off-target transcripts are identified by GESS based on direct analysis of primary screening data. The input file type in GESS was chosen as ‘input file type contains only active RNAi reagents’, the region was 3′UTR and the built-in reference database for humans was used. Both guide and passenger strands of siRNAs were analyzed separately for seed-dependent off-target. Finally, both guide and passenger strands of siRNAs were evaluated for the presence of immunostimulatory motifs ‘GUCCUUCAA’ [34] and ‘UGUGU’ [35]. The presence of the cytotoxic motif ‘UGGC’ was also tested [36].

### Target site accessibility & RNA duplex thermodynamics prediction by S-fold

The siRNA application module of S-fold (https://sfold.wadsworth.org/cgi-bin/sirna.pl) was used to predict target site accessibility and differential stability of siRNA duplex ends (DSSE). The target sequences of the siRNAs provided by siDirect2.0 were used for the prediction. The siRNAs predicted by S-fold after applying all S-fold filtering criteria were matched with the selected siRNAs. Several siRNA parameters are provided by S-fold, including target site accessibility score, DSSE, antisense siRNA binding energy, average internal stability at the cleavage site (AIS) and total stability of the siRNA duplex.

### Secondary structure determination & guide strand–target region interaction

The secondary structure of the guide strand of the siRNA and target site as well as their free energy of folding was determined using the MaxExpect program of the RNAstructure web server (https://rna.urmc.rochester.edu/RNAstructureWeb/). Higher free energy of folding denotes less chance of folding. The interaction between the guide strand and the target sequence was observed with the DuplexFold program of the RNAstructure web server. The free energy of binding is also calculated by DuplexFold. The more negative the free energy of binding, the stronger the binding.

### Docking of the siRNAs with AGO2

To perform molecular docking of the predicted siRNAs with AGO2, the authors first predicted the tertiary structure of the receptor and ligand molecules. For modeling of a complete AGO2 protein structure, homology modeling was performed using SWISS-MODEL (https://swissmodel.expasy.org/interactive) based on the crystal structure template of RNA-bound human AGO2 protein (Protein Data Bank [PDB] identifier: 4Z4D) [37,38]. The model was further refined using GalaxyRefine (http://galaxy.seoklab.org/cgi-bin/submit.cgi?type=REFINE) [39], and the quality of the finally selected model was assessed using ERRAT and Structure Assessment of SWISS-MODEL [38,40].

For the siRNA structure prediction, BIOVIA Discovery Studio 2021 was used. Here the RNA structure bound to the AGO2 crystal structure (PDB identifier: 4Z4D) was considered the template. The template RNA strand was mutated to model the targeted siRNA strands so that the conformational similarity to the actual model was maintained. Following structure prediction, the geometry of the resulting structure was optimized using Avogadro software [41]. Here Merck molecular force field 94 was used, and the parameters were set to steepest descent algorithm, 1000 steps and 10e-7 convergence.

**Figure 1.**
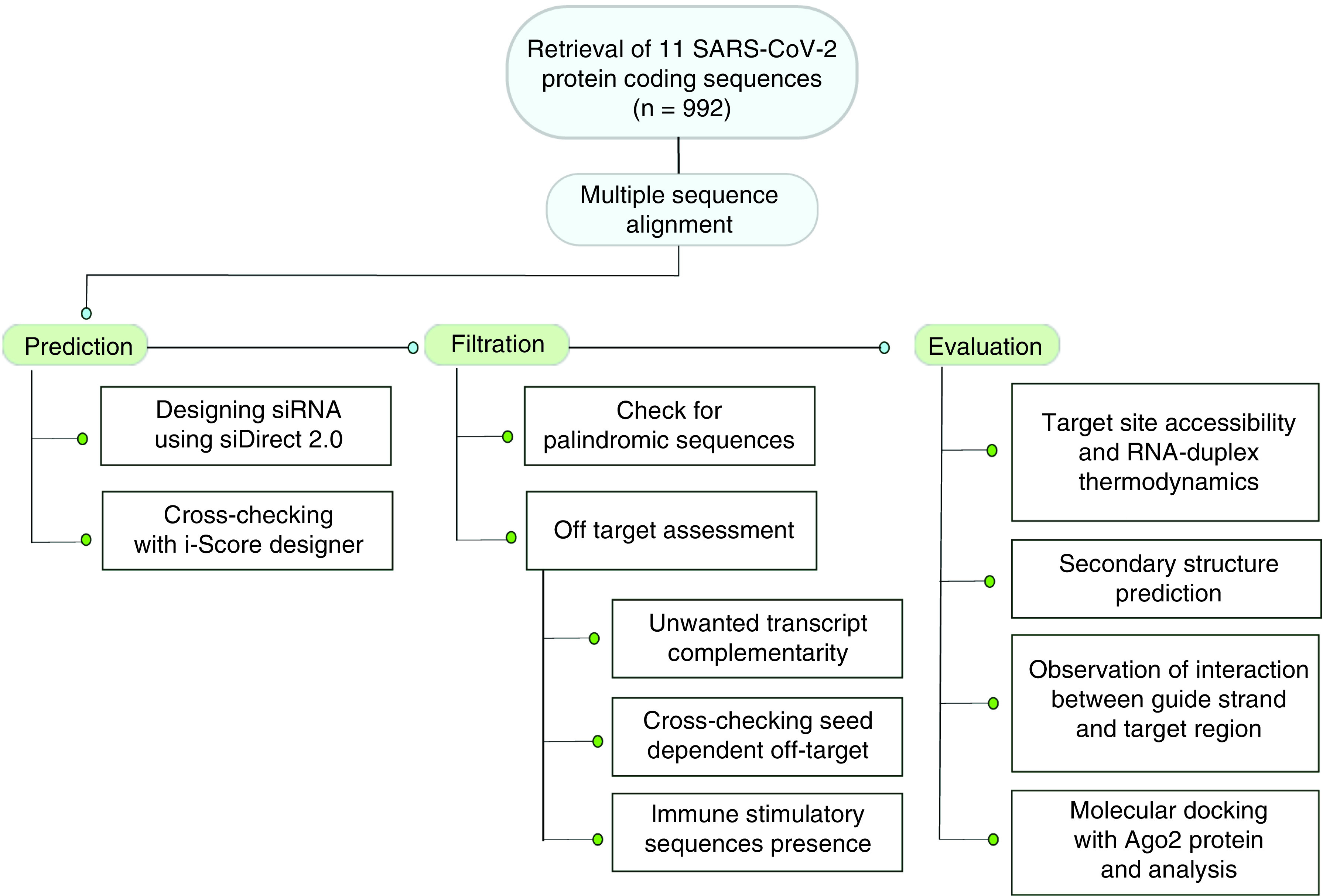
Graphical workflow of the methodology.

Finally, the predicted AGO2 structure and siRNA structures were docked using the HDOCK server (http://hdock.phys.hust.edu.cn/) [42]. The docking with AGO2 was performed in two ways: the dssiRNA structures were docked and the guide strands of the siRNAs were docked. The HDOCK server performed protein–RNA docking based on a hybrid algorithm of template-based modeling and predicted AGO2-RNA complexes along with the docking energy scores. The complexes with lower energy scores and closest siRNA binding position to the template were selected and visualized. The flow of the present study, from retrieval of sequences to docking, is demonstrated in Figure 1.

## Results

### Retrieval of nucleotide & protein sequences of SARS-CoV-2

The metadata consisted of 214,257 retrieved sequences from 1 September 2020 to 6 May 2021 (based on collection date, database accessed on 06/05/21). A total of 601 sequences were excluded, as the specimen collection month was not mentioned. A total of 116,771 sequences had accession numbers starting with ‘FR’, ‘HG’, ‘LC’, ‘LR’, ‘OA’, ‘OB’, ‘OC’ or ‘OD’, and they were excluded as well (Supplementary File 2). Thus, the final list comprised 96,885 (214257-116771-601) sequences. All 18 sequences from May 2021 were considered in this study for analysis. A total of 989 accession numbers of SARS-CoV-2 sequences were obtained from the final list after randomly selecting 1% of the sequences from each month (except May 2021). Pango lineages were assigned for 115 sequences that did not have an assigned lineage. The list of 989 accession numbers of SARS-CoV-2 sequences did not contain the lineage B.1.617 or its sublineages. There were 24 B.1.617 sublineages (19 B.1.617.1 and five B.1.617.2). To include the B.1.617 sublineages in the study, a total of 1% of the sequences were randomly selected from 24 sequences using the sample_n() function of the Dplyr package in R. Finally, a total of 992 (989 + 3) sequences were used for retrieval of CDSs of the 11 SARS-CoV-2 proteins corresponding to a particular nucleotide sequence from the virus portal. The number of sequences taken from each month is presented in Supplementary File 2. Of the 992 SARS-Cov-2 nucleotide sequences, 359 did not have the CDSs for the ORF8 protein. MW46883 and MW938052 did not have the CDSs for the ORF10 protein and MW550174 did not have the CDSs for the ORF7b protein. The number of CDSs for each protein is also given in Supplementary File 2.

The Pango lineage distribution of all 992 sequences is given in Supplementary File 1. The number of sequences taken from each Pango lineage is also given in Supplementary File 1. The variants B.1.1.7, B.1.351, P.1, B.1.427, B.1.429, B.1.526, B.1.526.1, B.1.525, P.2 and B.1.617.1 had a respective 358, two, eight, 21, 40, 41, 18, three, seven and three SARS-CoV-2 nucleotide sequences.

### Extraction of conserved regions from MSA files of CDSs

Conserved regions from leader protein, 3C-like proteinase and RdRp were extracted instead of the entire ORF1ab. The conserved regions extracted from CDSs for the 13 proteins are presented in Supplementary File 2. No conserved regions (length ≥21) were found for ORF7a and ORF8.

### Target-specific siRNA prediction

A total of 12 siRNAs (S = 1; E = 1; RdRp = 10) that were predicted by siDirect 2.0 followed all three rules (Ui-Tei, Reynolds and Amarzguioui) and had a seed duplex Tm <21.5°C and GC content 30–64%. Of these, eight siRNAs (RdRp = 8) were found to overlap with predicted siRNAs (identified through i-Score Designer), having a rank of 1 in all three algorithms (s-Biopredsi, i-Score and DSIR).

### Filtering siRNAs with palindromic sequences & off-target assessment of siRNAs

A total of seven of eight siRNAs did not have a palindromic sequence in the guide and passenger strands. These seven siRNAs were further analyzed for off-target assessment. Only two of seven siRNAs had <78% query coverage for both guide and passenger strands. The GESS results for the seed-dependent off-target for the guide and passenger strands of these siRNAs were not significant by Fisher's exact test (p > 0.05), Bonferroni, Bonferroni step-down (Holm) correction and Benjamini and Hochberg methods. The immunostimulatory motifs were not present in the guide and passenger strands of these two siRNAs (Table 1).

**Table 1.** Most potent predicted siRNAs along with their different characteristics.

Alias	Conserved region position in RdRp	Target sequence location within conserved region	siRNA target within conserved region	Guide (antisense) strand 5′->3′	Passenger (sense) strand 5′->3′	GC content (%)	Seed duplex Tm (°C)		Free energy of folding for target sequence	Free energy of folding for guide strand	Free energy of binding
							Guide	Passenger			
S1	2232–2273	15–37	TGCGTAAACATTTCTCAATGATG	UCAUUGAGAAAUGUUUACGCA	CGUAAACAUUUCUCAAUGAUG	33.33	18.1	14.7	1.7	1.8	-31.4
S2	2611–2651	13–35	CAGGAGTATGCTGATGTCTTTCA	AAAGACAUCAGCAUACUCCUG	GGAGUAUGCUGAUGUCUUUCA	42.86	19.2	20.3	1.2	1.8	-35.9

Guide and passenger strands of predicted siRNAs along with their GC content, seed duplex Tm and free energy of folding and binding.

GC: Guanine–cytosine; Tm: Melting temperature.

### Target accessibility & RNA duplex thermodynamics prediction by S-fold

S1 had a target site accessibility score of 8, whereas S2 had a target site accessibility score of 7. DSSE in kcal/mol was 0.8 for S1 and 5.3 for S2 [43]. AIS in kcal/mol was -5.7 kcal/mol for S1 and -7.9 kcal/mol for S2. Both S1 and S2 had a total siRNA duplex score of 17, which passed the S-fold-imposed threshold of 12. All S-fold filter criteria (Supplementary File 3) for functional siRNA were fulfilled by these two siRNAs. Parameters obtained from S-fold output for the two finally selected siRNAs are given in Supplementary File 4.

### Secondary structure determination & guide strand–target region interaction

**Figure 2.**
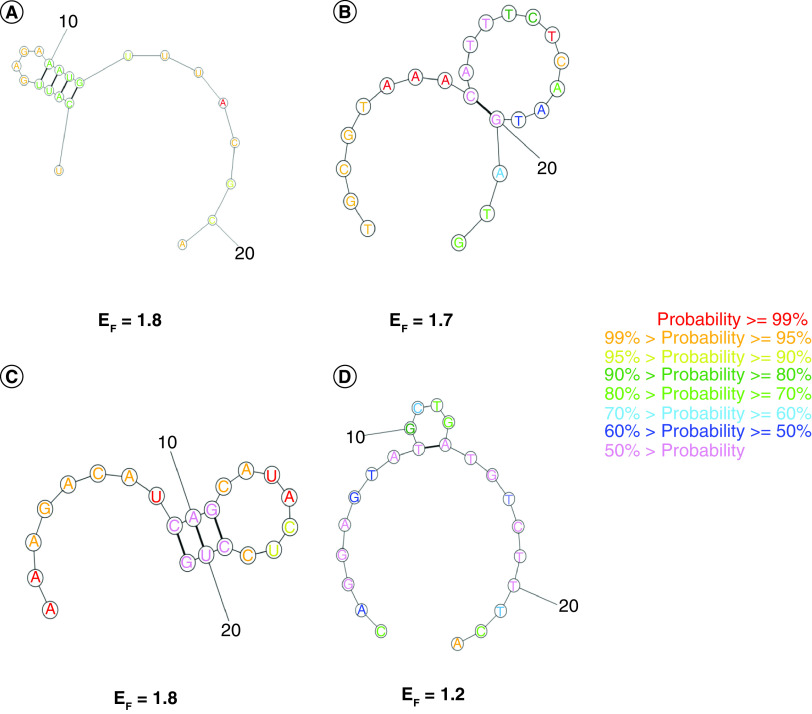
Secondary structures of the guide strands and target sequences of both effective siRNAs (S1 and S2) along with their free energy of folding. **(A)** Secondary structure of the guide strand of S1. **(B)** Secondary structure of the target sequence of S2. **(C)** Secondary structure of the guide strand of S2. **(D)** Secondary structure of the target sequence of S1.

**Figure 3.**
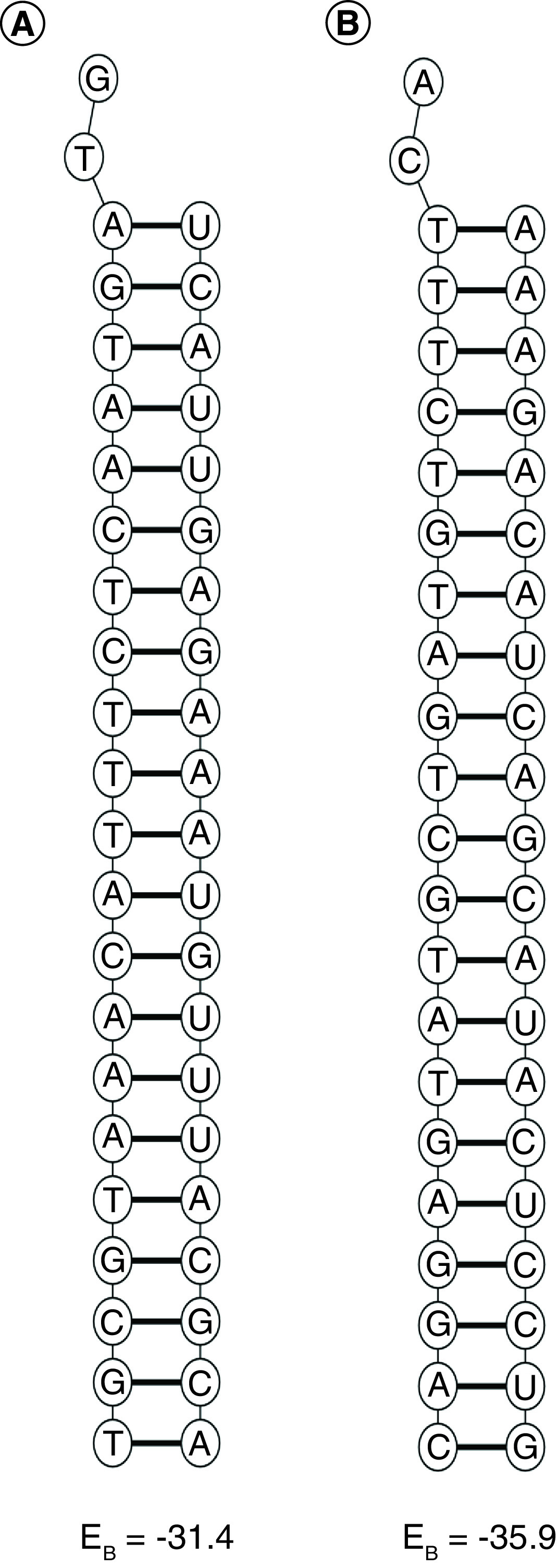
Structures of interaction between guide strands and target sequences of both effective siRNAs (S1 and S2) along with their free energy of binding. **(A)** Structures of interaction between guide strand and target sequence for S1. **(B)** Structures of interaction between guide strand and target sequence for S2.

The target site of S1 and the guide strand of S1 had free energy of folding values of 1.7 and 1.8, respectively The target site of S2 and the guide strand of S2 had free energy of folding values of 1.2 and 1.8, respectively (Table 1). The secondary structures of the target sites and guide strands of S1 and S2 are shown in Figure 2. The free energy of binding between the guide strand and target sequence was -31.4 for S1 and -35.9 for S2. The structures of binding between the guide strands and target sequences for S1 and S2 are shown in Figure 3.

### Molecular docking of AGO2 protein & predicted siRNA guide strands

The SWISS-MODEL-predicted structure of AGO2 was refined by GalaxyRefine, and the refined final structure quality was evaluated. This process helped to predict the missing residue conformation of the template structure. The ultimately selected structure possessed an ERRAT score of 94.34%, and the Ramachandran favored area was 98.92%, with 0.24% outliers. Moreover, the MolProbity and QMEAN scores were 1.32 and 0.34, respectively.

**Figure 4.**
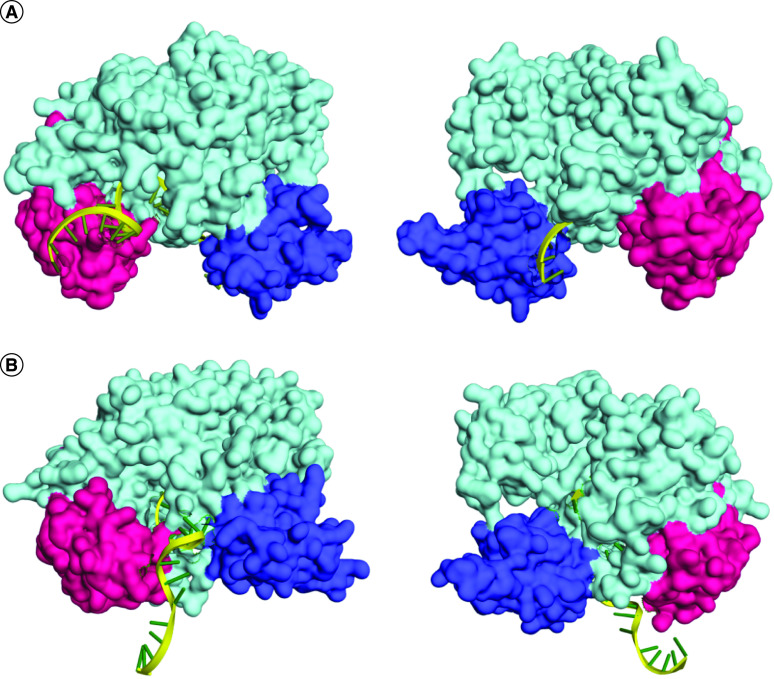
Molecular docking of siRNA guide strands and AGO2 protein. Docked structures of S1 siRNA guide strand **(A)** and S2 siRNA guide strand **(B)** with the AGO2 protein. The PAZ, MID and PIWI domains of the surface view structures of AGO2 are colored purple, pink and light green, respectively. The rest of the protein is colored light blue. The siRNA guide strands are colored yellow. For both complexes, two opposite views (front and rear) are presented.

Following the modeling of AGO2 protein, the authors performed docking with S1 and S2 siRNA guide strands. The best docking complexes were screened manually to detect placement of the RNA strands inside the PAZ and MID domains in the binding cavity (similar to the PDB 4Z4D complex). Next, based on the docking energy score, the best docked complexes were selected. Here the docking energy scores of the S1 guide strand–AGO2 and S2 guide strand–AGO2 complexes were -295.37 and -288.52, respectively (Figure 4). In analyzing these complexes, three kinds of interactions were observed between the receptor and the siRNA strands. For the S1 guide strand–AGO2 and S2 guide strand–AGO2 complexes, there were nine electrostatic bonds, 17 hydrogen bonds and four hydrophobic bonds and 11 electrostatic bonds, 16 hydrogen bonds and three hydrophobic bonds, respectively (Supplementary File 5).

**Figure 5.**
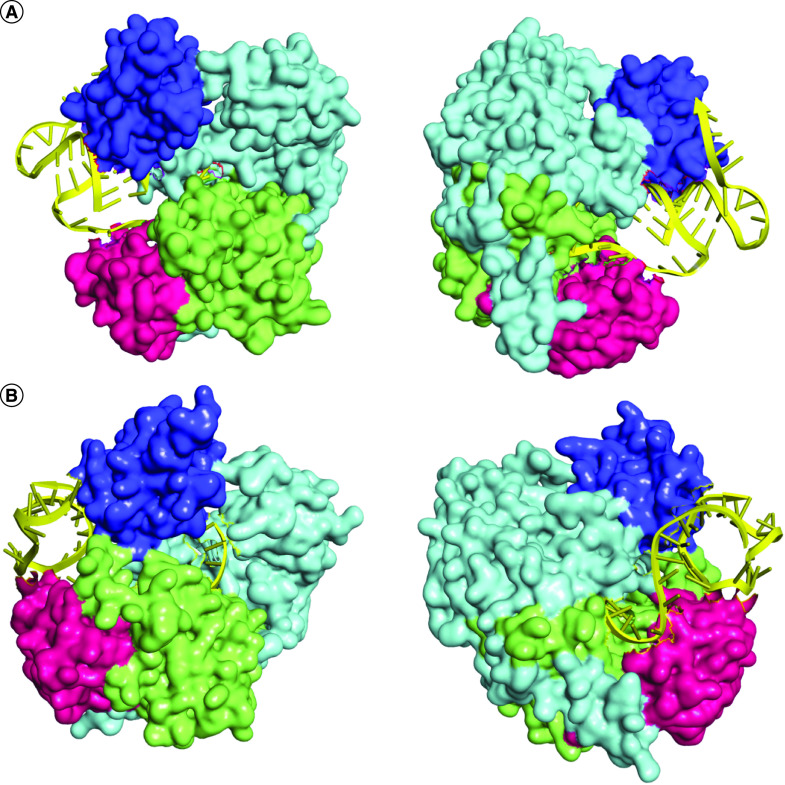
Molecular docking of dssiRNA and AGO2 protein. Docked structures of S1 dssiRNA **(A)** and S2 dssiRNA **(B)** with the AGO2 protein. The PAZ, MID and PIWI domains of the surface view structures of AGO2 are colored purple, pink and light green, respectively. The rest of the protein is colored light blue. The siRNAs are colored yellow. For both complexes, two opposite views (front and rear) are presented.

After this, to observe the docking interactions of dssiRNAs with AGO2, docking of the AGO2 receptor with S1 and S2 dssiRNAs was performed. Here the complexes demonstrated docking energy scores of -405.71 and -308.51, respectively, and three kinds of bonds between the protein and dssiRNA strands (Figure 5, Table 2 & Supplementary File 6).

**Table 2.** Number of different types of bonds between AGO2 receptor and dssiRNAs.

siRNA	Strand	Hydrogen bonds (n)	Electrostatic bonds (n)	Hydrophobic bonds (n)	Total bonds (n)
S1	Guide	13	16	2	31
	Passenger	3	4	-	7
S2	Guide	16	10	3	29
	Passenger	4	5	-	9

Number of hydrogen bonds, electrostatic bonds and hydrophobic bonds between AGO2 receptor and guide and passenger strands of siRNAs.

## Discussion

In this study, *in silico* methods were adopted to predict potential siRNAs against COVID-19. This study is unique in two important ways: firstly, sequences have been retrieved on the basis of sample collection date representing the strain of SARS-CoV-2 circulating in a particular month and secondly coding sequences of 13 SARS-CoV-2 proteins corresponding to the selected nucleotide sequences were used for siRNA prediction. The siRNAs were predicted using conserved sequences of SARS-CoV-2 comprising variants of interest and concern. As a result, the predicted siRNAs are thought to be effective against all variants included in the study.

The rules of Ui-Tei, Reynolds and Amarzguioui were derived from experimental evidence to facilitate siRNA prediction. The first-generation algorithms for siRNA design include these empirical rules. With the advent of Huesken's dataset of 2431 randomly selected siRNAs targeting 34 different mRNAs, many second-generation, machine-based algorithms have been developed for siRNA prediction. BIOPREDsi by Huesken *et al.* is based on an artificial neural network [31]. DSIR and i-Score are based on a linear regression model [32,33]. The S-Biopredsi score calculated by i-Score Designer has a 0.998 correlation coefficient with BIOPREDsi and hence produces almost identical results. The second-generation algorithms produce similar results in predicting functional and effective siRNAs [32,44]. Second-generation algorithms are more reliable, as they are developed using large datasets. In this study, the authors predicted siRNAs that gave the best result in both generations of algorithms. The predicted siRNAs (S1, S2) fulfilled all three rules (Ui-Tei, Reynolds and Amarzguioui) and had a rank of 1 in s-Biopredsi, i-Score and DSIR. The Ui-Tei class was Ia for both siRNAs, which meant that the siRNAs fulfilled all the rules of Ui-Tei [26]. Reynolds, Amarzguioui, s-Biopredsi, i-Score and DSIR were also high, which suggested that the predicted siRNAs were expected to be highly effective (Supplementary File 4).

Elbashir *et al.* reported 21-nucleotide siRNAs as the most potent siRNAs [45]. siRNAs designed by siDirect are 21 nucleotides in length. The Tm calculated by siDirect helps to minimize the seed-dependent off-target effects that might occur between the seed region (two to eight nucleotides from 5′ of siRNA guide strand) and 3′UTR of the unwanted mRNAs (miRNA-like off-target). Low Tm helps to minimize seed-dependent off-target effects. A Tm of 21.5°C (benchmark according to siDirect 2.0) helps to predict sequences which are almost seed-dependent off-target-free [46]. High GC content in siRNAs affects functionality, creating difficulty in RISC loading due to hindrance in duplex unwinding. Low GC content impacts siRNA effectiveness, as it may result in reduced affinity and specificity for the target sequence. The authors selected a GC content of 30–64% [29]. The presence of palindromic sequences may result in the formation of internal secondary structures, resulting in poor RISC loading.

BLASTN was used against the NCBI transcript reference sequences database for the prediction of perfect or near-perfect complementary to unwanted mRNAs. BLAST parameters were altered for precise alignment of small sequences. Although Tm minimizes seed-dependent off-target effects, GESS was used to check the seed-dependent off-target to ensure greater safety of the predicted siRNAs. No significant result was found in GESS, which is in agreement with the Tm result. The presence of the motif ‘GUCCUUCAA’ may lead to a nonspecific response as a result of interferon secretion [29]. Immunostimulatory sequences (‘GUCCUUCAA’ and ‘UGUGU’) and ‘UGGC’ were checked to prevent unwanted immune and cytotoxic reactions, respectively.

The siRNA module of S-fold was used for target site accessibility and RNA duplex thermodynamics. In siRNA module of S-fold, the thermodynamics indexes to infer siRNA duplex stability are based on Turner thermodynamics parameters [47,48]. RISC-mediated cleavage of mRNAs depends on target site accessibility in both *in vitro* and intracellular conditions [49]. Target site accessibility scores range from 0 to 8. S1 had a target site accessibility score of 8, whereas S2 had a target site accessibility score of 7. The high target site accessibility score for both siRNAs (S1 and S2) indicated good target binding. The difference between 5′end instability in the antisense strand and 5′end instability in the sense strand gives DSSE in kcal/mol. The siRNA strand with higher 5′end instability assembles with RISC (siRNA asymmetry). Therefore, to follow the asymmetry rule, DSSE should be >0 as the guide strand gets assembled with RISC. Assembling the passenger strand with RISC may result in ineffective and unwanted mRNA targeting. Functional siRNAs have higher instability at the 5′end of antisense strands [43,47].

Moreover, functional siRNAs have lower internal stability at positions nine to 14 (from 5′end of antisense strand) compared with nonfunctional siRNAs [43]. AIS in kcal/mol from the siRNA computes the average internal energy for positions nine to 14 in the antisense strand (counting from 5′end of antisense strand). S1 had a DSSE value of 0.8 and AIS value of -5.7. S2 had a DSSE value of 5.3 and AIS value of -7.9. Both S1 and S2 had an AIS value >-8.6 kcal/mol, as enforced by S-fold. A DSSE value >0 for both S1 and S2 depicts a higher chance of the guide strand assembling with RISC. The total siRNA duplex score is the sum of target accessibility score, duplex feature score and duplex thermodynamics score. The maximum total siRNA duplex score is 20. Duplex feature score is calculated based on the algorithm put forth by Reynolds *et al.* [27]. The duplex thermodynamics score has a maximum value of 2 and minimum value of 0 (1 point for DSSE >0 and 1 point for AIS >-8.6 kcal/mol). Both S1 and S2 had a high total siRNA duplex score of 17 and crossed the threshold of 12 at S-fold. The higher free energy of folding values for the guide strands and target sequences of both S1 and S2 clearly demonstrated that the guide strands and target sequences were less likely to fold. The high negative values for free energy of binding between the guide strands and target sequences of both S1 (-31.4) and S2 (-35.9) were in concordance with the high target site accessibility scores demonstrated with the siRNA module of S-fold. Previous studies targeted S [49], M [50], E [50,51], N [50], RdRp [51], 3C-like proteinase [52] and leader protein [53] of SARS-CoV for the silencing of further replication of the virus using an siRNA approach. In this study, the designed siRNAs (S1 and S2) targeted conserved regions within the RdRp protein. The designed siRNAs may inhibit the activity of RdRp by RISC-mediated cleavage of the coding sequences of RdRp.

Variants of concern (B.1.1.7, B.1.351, P.1, B.1.427 and B.1.429) and variants of interest (B.1.526, B.1.526.1, B.1.525, B.1.617, B.1.617.1, B.1.617.2, B.1.617.3 and P.2) were included in the study, together with other lineages. Completely (100%) conserved regions were used for the prediction of siRNAs, and hence the designed siRNAs are expected to be effective against a wide range of SARS-CoV-2 variants, including variants of concern and interest. Inclusion of CDSs of 13 SARS-CoV-2 proteins as well as variants of concern and interest makes this study unique compared with other studies involving siRNA design against SARS-CoV-2 [54–57]. Moreover, interactions between the siRNAs and AGO2 protein were evaluated by molecular docking.

AGO2 protein plays an important role in the proper functional execution of siRNAs. Two existing pathways are proposed to explain the phenomenon. It was first proposed that an ATP-dependent helicase unwinds the siRNA duplex and liberates the guide strand, and this guide strand further binds to AGO2 to exert its RNAi function [58]. Another proposal states that instead of receiving only the guide strand, the AGO2 protein binds the dssiRNA and then cleaves and liberates the passenger RNA strand. Thus, only the guide strand remains bound to it [59]. Either way, the formed guide RNA-AGO2 complex generates an active and mature RISC that attacks and cleaves mRNA transcripts complementary to the guide strand [60]. Thus, favorable docking of the guide siRNA strand or dssiRNA to the AGO2 is a crucial step in siRNA prediction. Human AGO2 protein possesses a bilobed structure that contains N-terminal (Asp53-Ser139), PAZ (Pro229-Val347), MID (Gly445-Pro580) and catalytic PIWI (Gln581-Ala859) domains. Here the PAZ and MID domains bind to the respective 3′ and 5′ ends of the siRNAs, and the PIWI domain bears RNase H-like slicer activity that facilitates RNA cleavage [61]. At the time of binding, the flexible PAZ domain moves toward the MID domain and assists the efficient anchoring of the 5′ and 3′ ends of the RNA to AGO2. However, as biphasic interaction of the 3′ end of the siRNAs with the PAZ domain is a must for RNAi activity, complexes with lower binding affinity are found to be better functioning. Thus, siRNAs with lower free energy and higher hydrogen bonding are associated with better RNAi activity [62,63]. Here electrostatic interactions play a minor role.

Upon exploration of the S1–AGO2 and S2–AGO2 guide RNA complexes, it was observed that the A11, G19, C20 and A21 residues of the S1 guide RNA bound to the PAZ domain with three hydrogen bonds (ARG286, ASP252, SER253) and two hydrophobic bonds (G19-VAL256, and C20-VAL256). By contrast, one hydrogen bond and two electrostatic bonds with ARG280 and LYS257 were observed involving the A1 and G11 residues of the S2 guide RNA. For the MID domain, seven hydrogen bonds and one electrostatic bond were observed for the S1–AGO2 complex and three hydrogen bonds and five electrostatic bonds were observed for the S2–AGO2 complex. Thus, for both siRNA guide strands, fewer bindings were observed for the PAZ domain compared with the MID domain, which might facilitate the dynamic bound and unbound states of siRNAs at the time of nuclease activity (Supplementary File 5).

Here both AGO2–guide strand complexes possessed an equal amount of bonds between AGO2 and the siRNAs, including the hydrogen bonds. The S1 and S2 AGO2–guide strand complex had 17 and 16 hydrogen bonds, respectively. Thus, based on the number of hydrogen bonds, both siRNA guide strands reflected an equal possibility of efficient RNAi function. However, in contrast to S2, the S1 guide strand–AGO2 complex had a higher docking energy score. In addition, visualization of the complexes showed that the S1 guide strand was completely encapsulated in the AGO2 cavity in a manner more similar to the PDB 4Z4D structure than the S2–AGO2 complex (Figure 4). This may indicate possible better performance than S2.

As another mechanism (in addition to the guide strand–AGO2 binding mechanism) has been proposed in which dssiRNA binds to AGO2 protein first and then the passenger strand gets cleaved, in this study, the authors also evaluated the AGO2–dssiRNA docking properties. Here it was observed that S1 and S2 dssiRNAs form 38 bonds, including hydrogen, electrostatic and hydrophobic bonds, with AGO2 protein, but in each case, the number of bonds for the guide strands was much higher than that observed for the passenger strands (Table 2 & Supplementary File 6). This indicated that the passenger strands were much more likely to get cleaved and leave the complex. In each dssiRNA-AGO2 complex, it was observed that the guide strands formed a smaller number of bonds with the PAZ domain in comparison to the MID and PIWI domains. For the double-stranded S1–AGO2 complex, the number of bonds of the guide strands with PAZ, MID and PIWI was four, eight and 14, respectively; for the double-stranded S2–AGO2 complex, the number of bonds of the guide strands with PAZ, MID and PIWI was zero, nine and nine, respectively (Supplementary File 6). This indicated that the guide strands in the complex possess a facilitated property that helps them to maintain their dynamic states after the cleavage of passenger strands.

Vaccines serve as prophylactic measures against COVID-19. Thus, based on these results, it can be stated that both of the predicted siRNAs have the possible ability to show effective performance in RNAi and may be considered potential therapeutic candidates against COVID-19. The designed siRNAs have gone through robust and stringent filtration steps that suggest high effectiveness and safety. The designed siRNAs may serve as therapeutic approaches to combating the current pandemic. However, these siRNAs need to be validated *in vitro* and *in vivo* for this purpose.

## Conclusion

The global SARS-CoV-2 pandemic has proven to be a great threat to humankind. siRNA-induced RNAi may play a critical role in the treatment of SARS-CoV-2. Two siRNAs that meet all the stringent and robust filtering criteria were predicted using computational and bioinformatics methodologies using the conserved regions of the coding sequences of 13 SARS-CoV-2 proteins corresponding to 992 SARS-CoV-2 sequences. However, further *in vitro* and *in vivo* studies must be done to confirm their actual therapeutic efficacy.

**Summary points**

- The emergence of new COVID-19 variants is making it difficult to combat this obstinate pathogen, and the high rate of infection and mortality underlines the urgent need for therapeutic intervention.
- The authors aimed to predict exogenous siRNAs that can inhibit viral transcription, replication and assembly through RNAi.
- In this study, conserved sequences of nucleotide coding regions of 11 SARS-CoV-2 proteins from a list comprising 96,885 sequences were used to construct siRNAs that will inhibit the life cycle of viral strains, including variants of concern and interest.
- Two siRNAs (S1: 5′-UCAUUGAGAAAUGUUUACGCA-3′ and S2: 5′-AAAGACAUCAGCAUACUCCUG-3′) against SARS-CoV-2 RdRP passed through the stringent filtering process and showed good binding characteristics with their targets, including AGO2 protein.
- As a result of the robust filtering process applied, the designed siRNAs are predicted to be highly effective and safe.
- The designed siRNAs are expected to inhibit viral replication and transcription in a wide range of coronavirus variants, including variants of concern (B.1.1.7, B.1.351, P.1, B.1.427, B.1.429) and variants of interest (B.1.526, B.1.526.1, B.1.525, B.1.617, B.1.617.1, P.2), as they were designed using the conserved regions.
- The predicted siRNAs are expected to be effective in the RNAi mechanism against SARS-CoV-2 proteins and may be considered potential therapeutic measures following *in vitro* and *in vivo* validation.
